# Acknowledgement to Reviewers (2015–2016) and general information about *Parasite*

**DOI:** 10.1051/parasite/2017011

**Published:** 2017-03-21

## Abstract

The Editors provide a list of the 237 reviewers from 43 countries who assessed manuscripts for *Parasite* in 2015 and 2016 and thank them for their help. The rejection rate has been about 50% since 2013. The mean time from submission to final publication was 104 days in 2016. The Impact Factor of *Parasite* has risen from 0.822 (published in 2014) to 1.781 (published in 2016). All papers published in *Parasite* and its predecessor *Annales de Parasitologie Humaine et Comparée* since 1985 are freely available from the website of the journal.

## Abstract (fr)

Les Éditeurs scientifiques fournissent une liste des 237 évaluateurs, de 43 pays, qui ont revu des manuscrits pour *Parasite* en 2015 et 2016, et les remercient pour leur aide. Le taux de refus est approximativement 50 % depuis 2013. Le délai de publication, de la soumission à la publication finale, a été de 104 jours en 2016. Le facteur d’impact de *Parasite* a augmenté de 0.822 (publié en 2014) à 1.781 (publié en 2016). Tous les articles publiés dans *Parasite* et son prédécesseur *Annales de Parasitologie Humaine et Comparée* depuis 1985 sont disponibles gratuitement sur le site internet du journal.

The Editor-in-Chief would like to thank all the reviewers who have given so generously of their time to assess manuscripts submitted to *Parasite*. The success of the journal depends upon their care and competence and their conscientiousness is much appreciated.

For 2015 and 2016, our **237 reviewers from 43 countries** are listed below. Names are in alphabetical order. Some have reviewed more than one manuscript. Note that for 2013–2014, the list included 292 reviewers from 51 countries [1].

The rejection rate of *Parasite* has been about 50% since January 2013 and this list includes reviewers who evaluated papers which were not published.

## Publication speed

We are happy to be one of the fastest journals in the field: 104 days from submission to publication in 2016. Papers published in *Parasite* are final versions: we do not publish uncorrected manuscripts or preliminary versions. Once published in *Parasite*, papers are in PubMed generally within 24 h (Table 1).

**Table 1.** Mean times for publication of papers in *Parasite*.

Mean time	2013	2014	2015	2016
From submission to acceptance (days)	95	111	106	85
From acceptance to final publication (days)	14	15	17	19
From submission to final publication (days)	109	126	123	104

## Impact Factors and other metrics

The Impact Factor (IF) still continues to be the key metric used by the academic community as a measure of quality of a journal. We are happy to show that the IF of *Parasite* (Table 2) has greatly improved over the last few years, and our expectation is for it to increase again. We are thankful to our authors and reviewers for this exceptional achievement.

**Table 2.** Impact Factor of *Parasite.*

Year of publication of Impact Factor	2014	2015	2016
IF	0.822	1.092	1.781

In addition to the Impact Factor, other metrics are also becoming important. Academics and researchers are now evaluating not only the Journal as a complete entity, but also individual article metrics. Since 2015, *Parasite* has included *Altmetric* scores for all papers, including the papers published before this date. We are very pleased to see that many papers published in *Parasite* have received significant *Altmetric* scores, and have impressive download counts. All this information is visible for each paper from our website.

## World impact

All papers in *Parasite* are published in English. While we acknowledge the international role of English in the scientific community, we also respect other cultures and communities. In addition to the English abstract, all papers have a French abstract and on various occasions we have also added an Arabic or a Chinese abstract, and we are open to suggestions for future papers.

While the proportion of French authors is stable, we have experienced a tremendous increase in submission of papers from China, from 2% of submitted papers in 2013 to 18% in 2016. Papers from *Parasite* have been downloaded from virtually every country on the planet, with more than 240,000 downloaded from our website in 2016, plus many more from other platforms.

## Tradition, modernity and open-access

*Parasite* is the successor to *Annales de Parasitologie Humaine et Comparée*, which was published from 1923 to 1993. We have now scanned and uploaded all papers published since 1985, and we intend to do more this year. Of course, all these papers are free to download with no pay-wall: we do not charge our readers $40 for a paper published 30 years ago!

All papers published in *Parasite* are full gold open-access papers with free immediate access for all to read. They are also available from other platforms such as PubMed Central, Europe PubMed Central, DOAJ and Science Open. In addition to traditional texts and images, we can publish movies and other media. All papers are available in html, PDF and even ePub formats to be readable on mobile devices.

## List of reviewers in 2015 and 2016

Rob Adlard (Australia)
Ève Afonso (France)
Bulent Alten (Turkey)
Cristian A. Alvarez Rojas (Australia)
Samer Angelone-Alasaad (Switzerland)
Karim Aoun (Tunisia)
Andrea Apolloni (France)
Thomas Barth (Germany)
Christian Bauer (Germany)
Ian Beveridge (Australia)
Oleg Blagosklonov (France)
Peter Boag (Australia)
Franck Boué (France)
Nacim Bouheraoua (France)
Denis Boulanger (France)
Gilles Bourdoiseau (France)
Jérémy Bouyer (France)
Rodney A. Bray (United Kingdom)
Reginhaldo Brazil (Brazil)
Emanuele Brianti (Italy)
Alessandro Broglia (Italy)
Joanna Browne (Australia)
Magdaléna Bruňanská (Slovakia)
Nophawan Bunchu (Thailand)
Jacques Cabaret (France)
Oscar Cabezón (Spain)
Ermanno Candolfi (France)
Luis Cardoso (Portugal)
Yannick Caron (Belgium)
Sócrates C.H. Cavalcanti (Brazil)
Ivan Cepicka (Czech Republic)
Gabriela Certad (France)
Piotr Ceryngier (Poland)
Amira Chaabane (Tunisia)
Qijun Chen (China)
Jia-Xu Chen (China)
Jia Chen (China)
Eirini Christaki (Greece)
Theresa Coetzer (South Africa)
Simone Cohen (Brazil)
Douglas Colwell (Canada)
Melissa Conrad (United States)
Barbara Conti (Italy)
Michał Czopowicz (Poland)
Inácio Domingos da Silva Neto (Brazil)
Marie-Laure Dardé (France)
Arwid Daugschies (Germany)
Isaure de Buron (United States)
Reginald De Deken (Belgium)
Johan F. De Jonckheere (Belgium)
Harry De Koning (United Kingdom)
Laurence Delhaes (France)
Jérôme Depaquit (France)
Peter Deplazes (Switzerland)
Angela Di Cesare (Italy)
María Celina Digiani (Argentina)
Bilal Dik (Turkey)
Isabelle Dimier-Poisson (France)
Brent Dixon (Canada)
Olgica Đurković-Đaković (Serbia)
Stephen Doggett (Australia)
Philippe Dorchies (France)
Gilles Dreyfuss (France)
Jitender Dubey (United States)
Jean Dupouy-Camet (France)
Olivier Duron (France)
Itziar Estensoro (Spain)
Bart Everts (Netherlands)
Jorge Falcón-Ordaz (Mexico)
Alessandra Falleni (Italy)
Anna Faltýnková (Czech Republic)
Vinicios Ferreira-de-Freitas (United States)
Hubert Ferté (France)
William Font (United States)
Elena Forte (Italy)
Michel Franc (France)
José Ramon Franco (Switzerland)
Claire Fuller (United Kingdom)
Terry Galloway (Canada)
André Garcia (Benin)
Scott Gardner (United States)
Claudio Genchi (Italy)
Dirk Geysen (Belgium)
Mohamed Gharbi (Tunisia)
Lynda Gibbons (Australia)
David Gibson (United Kingdom)
Pooria Gill (Iran)
David González-Solís (Mexico)
Bruno Gottstein (Switzerland)
Philippe Grellier (France)
Frédéric Grenouillet (France)
Norman Grim (United States)
Zemao Gu (China)
Ankita Gupta (India)
Nabil Haddad (Lebanon)
Ali Halajian (South Africa)
Sarah Hamer (United States)
Gabriel Hamer (United States)
Clare Hamilton (United Kingdom)
Majid Fasihi Harandi (Iran)
Epco Hasker (Belgium)
Louise Heaton (United Kingdom)
Martin Heyworth (United States)
Hervé Hoste (France)
Aurore Hounto (Benin)
Sandrine Houzé (France)
Shaomin Hu (United States)
Sung-Tae Hung (Korea)
Seth Irish (United States)
Tadashi Itagaki (Japan)
Armando Jardim (Canada)
Minjun Ji (China)
Quentin Jossart (Belgium)
Jean-Lou Justine (France)
Zakaria Kambris (Lebanon)
Saumyakanti Khatua (India)
Marta Kicia (Poland)
Muza Kirjušina (Latvia)
Jovin Kitau (Tanzania)
Seiki Kobayashi (Japan)
Ismail Soner Koltas (Turkey)
Jan Kopecký (Czech Republic)
Alexei Kostygov (Czech Republic)
Michail Kotsyfakis (Czech Republic)
Michael Kristensen (Denmark)
Delane Kritsky (United States)
Katrin Kuhls (Germany)
Karien Labuschagne (South Africa)
Gordon Langsley (France)
Claire Laugier (France)
Antti Lavikainen (Finland)
Claudio Lazzari (France)
Sylvie Lecollinet (France)
Xiangrui Li (China)
Quan Liu (China)
Xiao-gang Liu (China)
Gabriella Lo Verde (Italy)
Philippe Loiseau (France)
Jacob Lorenzo-Morales (Spain)
Hannelore Lotter (Germany)
Eugene Lyons (United States)
Sutherland Maciver (United Kingdom)
Helena Maltezou (Greece)
Francesca Mancianti (Italy)
Tanguy Marcotty (Belgium)
Davis Mark (United States)
Justin Masumu (Congo)
Chris McAllister (United States)
Catherine McCann (United Kingdom)
Emily McDonald (United States)
Antonio Menezes da Silva (Portugal)
Jean Menotti (France)
Alessandro Minelli (Italy)
David Modrý (Czech Republic)
Paul Monis (Australia)
Scott Monks (Mexico)
José Alberto Montoya-Alonso (Spain)
Serge Morand (France)
Luciano Andrade Moreira (Brazil)
Ralf Mueller (Germany)
Beat Mülhaupt (Switzerland)
John-Paul Mutebi (United States)
Changshen Ning (China)
Matthew Nolan (United Kingdom)
Francesco Nugnes (Italy)
Emiliano Ocampo (Argentina)
Takahiro Ohnishi (Japan)
Susumu Ohtsuka (Japan)
Laor Orshan (Israel)
Domenico Otranto (Italy)
Elias Papadopoulos (Greece)
Carine Paraud (France)
Philippe Parola (France)
Steven Peck (United States)
Hervé Pelloux (France)
Gerardo Pérez Ponce de León (Mexico)
Bernard Pesson (France)
Klára Petrželková (Czech Republic)
Kurt Pfister (Germany)
Davies Pfukenyi (Zimbabwe)
Larisa Poddubnaya (Russian Federation)
Bruno Polack (France)
Robert Poulin (New Zealand)
Edoardo Pozio (Italy)
Iva Přikrylová (Czech Republic)
Heather Proctor (Canada)
Nicolas Puillandre (France)
Stéphane Ranque (France)
Eva Řehulková (Czech Republic)
Klaus Reinhardt (Germany)
Mickael Riou (France)
Joao Rodrigues (Portugal)
Brice Rotureau (France)
Urmas Saarma (Estonia)
Oscar Salomon (Argentina)
Vittorio Sambri (Italy)
Rodrigo Sanabria (Argentina)
Claudia Santos (Brazil)
Roy Sawyer (United States)
Tomas Scholz (Czech Republic)
Roshanak Tolouei Semnani (United States)
Jeffrey Shaw (Brazil)
Sara Shayan (Iran)
Myeong Heon Shin (Korea)
Gustave Simo (Cameroon)
Tina Skinner-Adams (Australia)
Nico Smit (South Africa)
Todd Smith (Canada)
John Soghigian (United States)
Philippe Solano (France)
Tatiana Sulesco (Moldova)
Marc Thellier (France)
José Tort (Uruguay)
Simon Tragust (Germany)
Tomáš Tyml (Czech Republic)
Maarten Vanhove (Belgium)
Isabelle Villena (France)
Philippe Vincendeau (France)
Petr Volf (Czech Republic)
Dominique Angèle Vuitton (France)
Hai-Long Wang (China)
Lesley Warner (Australia)
Marion Wassermann (Germany)
Richard Wilkerson (United States)
Thierry Wirth (France)
Samanta Xavier (Brazil)
Masahiro Yamamamoto (Japan)
Tingbao Yang (China)
Yurong Yang (Australia)
Aneta Yoneva (Bulgaria)
Hak Sun Yu (Korea)
Elżbieta Żbikowska (Poland)
Galina Zemstova (United States)
Longxian Zhang (China)
Nian-Zhang Zhang (China)
Wenbao Zhang (China)
Hejun Zhou (China)
